# Continuous-time modeling of the multivariate relationships between physical activity levels and stationary time in preschool-aged children: an investigation of the ActivityStat hypothesis

**DOI:** 10.1186/s44167-023-00039-z

**Published:** 2023-12-06

**Authors:** Carminda Goersch Lamboglia, Geralyn R. Ruissen, Nicholas Kuzik, Valerie Carson, John C. Spence

**Affiliations:** 1 Faculty of Kinesiology, Sport, and Recreation, University of Alberta, Edmonton, AB T6G 2H9 Canada; 2 Children’s Hospital of Eastern Ontario Research Institute, Ottawa, ON Canada

**Keywords:** Activity compensation, Movement, Sedentary behaviour, Accelerometery, Child

## Abstract

**Background:**

The ActivityStat hypothesis proposes that an increase or decrease in physical activity (PA) greater than a certain set point activates behavioural and/or energy compensatory responses to maintain a stable level of total energy expenditure. Few studies have tested this hypothesis in children and even fewer have focused on young children. Therefore, the purpose of this study was to investigate the ActivityStat hypothesis by examining the presence and timeframe of the relationships among PA levels and stationary time (ST) in preschool-aged children.

**Methods:**

A secondary analysis was performed on repeated measurement data (i.e., day-to-day activity) involving 98 preschool-aged children (age: 4.5 ± 0.7 years) in Edmonton, Canada. Participants were asked to wear an ActiGraph wGT3X-BT on the waist for 7 consecutive days to assess PA levels (i.e., light PA [LPA], and moderate-to-vigorous PA[MVPA]) and ST. Bayesian continuous-time structural equation modeling (CT-SEM) was used to examine the relationship between behaviours over time and the timeframe during which these relationships occur.

**Results:**

Each behaviour (i.e., LPA, MVPA, and ST) positively and meaningfully predicted itself at a later time. These relationships persisted up to 0.5 days later, at which point past behaviour no longer meaningfully predicted future behaviour. In contrast, no relationships were observed between the three behaviours.

**Conclusions:**

This is the first study to investigate the ActivityStat hypothesis using Bayesian CT-SEM in preschool-aged children. When simultaneously taking into account all dynamic relationships suggested by the ActivityStat, the findings fail to support the hypothesis.

## Introduction

To experience the health benefits of physical activity (PA), public health guidelines recommend preschool-aged children (under 5 years old) accumulate 180 min of PA daily, including at least 60 min of moderate-to-vigorous PA (MVPA), and limit time spent sedentary [1]. However, a majority of children have insufficient levels of PA and spend long hours in sedentary-related behaviours globally [2]. To date, efforts to produce sustained changes in health-enhancing PA and sedentary behaviour (SB) remain a formidable challenge [3]. Thus, new perspectives are needed to understand the mechanisms underpinning children's activity patterns. Biological factors, for instance, may also influence how individuals engage in PA but investigation in this area is scarce [4].

The ActivityStat hypothesis proposes that humans have biological mechanisms that regulate the extent to which individuals engage in PA [5]. It is hypothesized that an internal biological control center (i.e., hypothalamus) is activated when total energy expenditure reaches its set point triggering a cascade of compensatory mechanisms to return the system back to its steady state [4–6]. Specifically, an increase or decrease in PA greater than a certain set point activates behavioural and/or energy compensatory responses to maintain a stable level of total energy expenditure. This evolutionary trait keeps energy requirements in check while prioritizing and allocating energy to reproductive fitness and survival [6]. In studies involving children, both within- and between-days have been examined for evidence of behavioural compensation. For instance, within-day activity compensation was observed in children who had an active morning commute and then walked less throughout the day [7]. As for between-days compensatory effects, Ridgers et al. [8] showed that for every additional 10 min of MVPA on one day resulted in a reduction of 9.3 min of MVPA and 16.8 min of LPA the following day in children 8–11 years of age. Likewise, associations between sitting and PA both within- and between-days show that children may compensate for increased sitting, standing, and stepping [9]. However, other empirical studies and systematic reviews do not support compensatory mechanisms in this population [5, 10, 11]. Thus, the ActivityStat presents an intriguing area for further study. Additionally, preschool-aged children are underrepresented in studies examining compensatory mechanisms of PA and SB, suggesting a new avenue of research.

A lack or inadequate alignment between theory (i.e., ActivityStat), methods, and statistical modelling may explain inconsistent findings in the literature [12]. Studies investigating the ActivityStat hypothesis have mainly used traditional longitudinal multilevel models (e.g., hierarchical linear models, mixed models) [8, 11, 13]. These include the analysis of static mechanisms using univariate approaches focusing on the relationship among the constructs in one direction or the other. For example, to understand how a change in individuals’ MVPA at one point results in changes in SB at a subsequent point in time, separate analyses examining the relationship of MVPA on SB, and the relationship of SB on MVPA are typically conducted. However, without accounting for autoregressive effects (i.e., the relationship within the same variable over time) of all study variables and the reciprocal cross-lagged effect (i.e., the relationship between variables over time) within the same analysis, the observed lagged effects will be artificially inflated [14]. Therefore, findings for the ActivityStat hypothesis that are based on traditional longitudinal multilevel models should be interpreted with caution.

Instead, dynamic modelling methods are more appropriate for examining the ActivityStat hypothesis for several reasons. Specifically, such modelling methods: (1) account for multivariate reciprocal relationships between the constructs studied; (2) allow the analysis of variables that act both as predictor and outcome variables; and (3) include testing of a system as a whole, with all relevant constructs in one model [15]. For instance, continuous-time structural equation models (CT-SEM) treat dynamic processes as a function of the continuous variable of time. Thus, findings can be generalized to other timeframes providing a complete picture of how a phenomenon unfolds over time [16, 17]. Additionally, a novel advantage of CT-SEM is it can help answer research questions regarding the temporal specificity (i.e., timeframe) of when the behavioural compensation may occur (e.g., acute effect, between days, between multiple days).

Therefore, the current study aims to investigate the ActivityStat hypothesis in preschool-aged children by using CT-SEM to (1) examine the presence of continuous-time multivariate relationships between PA levels (operationalized by LPA and MVPA) and SB (as represented by stationary time [ST]) [18], and (2) explore the temporal specificity underlying the multivariate relationships between PA levels and ST.

## Methods

### Participants and procedure

This study involved a secondary analysis of data derived from the Parent–Child Movement Behaviours and Pre-School Children’s Development project [19]. The original cross-sectional study recruited children, aged 3–5 years, and their families in Edmonton, Canada and surrounding areas through a program that aims to teach fundamental sport skills through play. A total of 131 children initially agreed to participate and data collection occurred from July to November 2018. For both the primary and secondary analysis, approval was granted by the University of Alberta research ethics board.

### Measures

LPA, MVPA, and ST were measured with ActiGraph wGT3X-BT accelerometers. Because this accelerometer is limited in reliably estimating body posture [20], the recommendation is to use ST to represent sedentary-related behaviors [18]. Specifically, ST refers to the time spent in “any waking behavior done while lying, reclining, sitting, or standing, with no ambulation, irrespective of energy expenditure” [18]. Participants were instructed to wear the accelerometer on an elastic belt on their right hip for 24 h a day over 7 days, except during water-based activities. The devices were programmed at 30 Hz for sampling frequency and the data were downloaded in 15-s epochs for both normal filter files and low frequency extension filter files. The normal filtered files were used to categorize children’s LPA (26–419 counts/15 s), MVPA (≥ 420 counts/15 s), and ST (≤ 25 counts/15 s). Days with valid wear time of at least 10 h/day (i.e., 600 min) and a minimum of 3 days represented a valid and reliable estimate for PA and ST via accelerometry [21], and non-wear time was removed (≥ 20 min consecutive 0 counts [22]). Minutes per day for LPA, MVPA, and ST were then included in the analysis. Though sleep is part of the activity spectrum [23], and should be considered when testing the ActivityStat hypothesis, its inclusion in the current analysis was not possible due to model complexity in CT-SEM. Regardless, the focus on PA and ST allows for comparisons with previous studies on the topic [7–9, 11].

### Statistical analysis

Accelerometry data were structured in a stacked format (i.e., long format) and a time index variable was created to identify each time-point (i.e., day, *t* = 0–6). Data were standardized, and grand-mean centered to facilitate model convergence. Bayesian CT-SEM uses distinct modeling allowing for more flexibility compared to traditional longitudinal multilevel models [16]. For example, Bayesian estimation methods are robust to moderate violations of the normality assumption [24]. Regardless, the shape of the distributions for the outcomes of interest were visually inspected and all appeared normal. Due to the use of a continuous-time framework for this study, missing data were considered missing at random. Specifically, it was interpreted as reflecting unequal time intervals between measurements [25]. Further, Bayesian CT-SEM does not impose any restriction on sample size and length of time series (i.e., minimum number of days or other time period/s) [16]. The models were fit using the ctsem package [26], which interfaces to Stan [27] in the R 4.2.1 environment [28]. We computed a CT-SEM model examining the multivariate dynamic system with reciprocal relationships between LPA, MVPA, and ST.

As recommended for Bayesian models, the default burn-in (50% of the chain [i.e., one sequence of randomly sampled values]), the default aggregation statistic (mean of the chain), and the default priors were used [16]. The model (i.e., LPA, MVPA, ST) was run using a NUTS (No U-Turn sampler) with four chains and 20,000 iterations per chain [29]. For the model convergence and statistic precision, the potential scale reduction factor $\widehat{R}$ and effective sample size (N_eff_) were reported [29]. A fully random effects model was first constructed, however, it showed problems with model fit. A random intercept only model was also performed presenting a better fit with an adequate model convergence and precision. The inclusion of the random intercept in the model allows variation in the intercept only resulting in similar parameters estimated in the model, thus accommodating stable individual differences, but not individual differences in the dynamic relationships. As the ActivityStat hypothesis proposes that these compensatory mechanisms occur within-individuals [5], marked differences in these compensatory dynamics between individuals were outside the scope of this study. Thus, the latter findings are presented here. Further details about the complete code and the fully random effects model output illustrating the pattern of results can be obtained from the authors.

The auto- and cross-effects parameters are of primary interest for answering the main research question and refer to the continuous-time parameters presented in the drift matrix. The auto-effects reflect the stability (or persistence) of the relationship within a behaviour over time. The cross-effects describe the reciprocal effects of one variable on the other. The autoregressive and cross-lagged effect terms refer to the discrete-time parameters. From these parameters, the time interval at which dynamic processes reach their peak effects and their maximum and minimum discrete-time coefficient time intervals were examined. Time-varying factors are statistically accounted for based on the stochastic element of our model (i.e., diffusion matrix) [16]. Time-invariant differences (i.e., age, gender) were accounted for through a random intercept [30, 31].

For interpreting the results, the parameter’s posterior mean (M) in relation to its posterior standard deviation (SD) and posterior 95% Bayesian credibility intervals (BCI) were examined. Meaningful results are interpreted when zero does not fall within the lower (2.5%) or upper (97.5%) limits of the BCI parameter. Evidence of continuous-time temporal relationship is indicated by meaningful auto- or cross-effect relationships between PA levels and ST. Finally, evidence of temporal specificity is indicated by the timeframe at which the autoregressive or the cross-lagged effects between PA levels and ST reach their peak effects. Dynamic processes were analysed as a function of the continuous variable of time with activity levels aggregated at the day level.

## Results

A total of 98 children met the minimum wear-time criteria with an average of 772 min per day (SD = 66.65, range 623.36–937.91) and 46% presented 7 days of valid accelerometer data. Boys represented 69.4% of the sample and the average age was 4.5 ± 0.7 years. The children engaged in an average 303.18 ± 49.09 min of LPA, 108.72 ± 40.29 min of MVPA, and 360 ± 72.59 min of ST per day.

The Bayesian estimation required approximately 20 RAM usage and 15.8 h of runtime for the LPA, MVPA, and ST model on an Intel i9-10900 T (4.60 GHz Turbo) CPU of a 64-bit Windows OS, with 64 GB RAM. All parameters had a minimum of 2270.19 effective samples and a Rhat ($\widehat{R}$) of 1.0, indicating adequate model convergence and precision. Based on the prediction model by Hecht and Zitzmann [24, 32] using *N* = 98, *T* = 7 (total number of time points), and standardized peak effect set to 0.01 (small effect), our post hoc analysis suggests we had a sufficient sample size to reliably estimate the continuous-time cross-lagged dynamics between LPA, MVPA, and ST (estimated “power” for standardized peak cross-lagged effect = 1.00).

Table 1 displays the posterior population means, standard deviations, and 95% Bayesian credibility intervals for the T_0_ mean parameters, continuous-time intercept (i.e., b), and T_0_ variance between LPA, MVPA, and ST. The T_0_ mean indicates the initial state (i.e., starting point) estimate for the outcomes. It shows to what extent the participants’ initial states tend to be higher or lower than their later states [16, 17]. A negative estimate indicates that the initial process was lower than future states, whereas a positive estimate indicates that the initial state was higher. For LPA, MVPA, and ST, no meaningful increase or decrease was observed in the overall level over time, with the 95% BCI encompassing 0 (LPA, M = 0.08, SD = 0.1, 95% BCI [− 0.12, 0.27]; MVPA, M = 0.16, SD = 0.11, 95% BCI [− 0.05, 0.38]; ST, M = − 0.18, SD = 0.1, 95% BCI [− 0.37, 0.02]). This means that LPA, MVPA, and ST fluctuated around a stationary, average level, during the observation period (i.e., 7 days). The continuous-time intercept represents the average process means for each outcome studied. Because they were grand-mean centered and standardized, they are not relevant for interpretation. The T_0_ variance, which refers to the asymptotic within-person covariance, indicated that all processes studied have similar variation over time (LPA, M = 0.98, SD = 0.07, 95% BCI [0.85, 1.13]; MVPA, M = 1.07, SD = 0.08, 95% BCI [0.93, 1.24]; ST, M = 0.98, SD = 0.07, 95% BCI [0.85, 1.13]).

**Table 1:** Means of estimated population distributions of the relationship between LPA, MVPA, and ST

Parameter		BCI					
		Est	SD	[2.5%, 97.5%]		Rhat	N_eff_
T_0_ mean	LPA	0.08	0.1	− 0.12	0.27	1	56,627.31
	MVPA	0.16	0.11	− 0.05	0.38	1	50,793.65
	ST	− 0.18	0.1	− 0.37	0.02	1	50,109.75
Continuous-time intercept	LPA	− 0.04	0.25	− 0.6	0.42	1	14,581.17
	MVPA	− 0.2	0.49	− 1.31	0.73	1	20,603.15
	ST	0.1	0.28	− 0.39	0.73	1	14,621.88
T_0_ variance	LPA	0.98	0.07	0.85	1.13	1	61,489.69
	MVPA	1.07	0.08	0.93	1.24	1	53,878.21
	ST	0.98	0.07	0.85	1.13	1	55,909.85
Auto-effects parameters	LPA	− 3.13	2	− 9.04	− 1.24	1	4717.66
	MVPA	− 6.11	2.79	− 12.23	− 1.95	1	8155.83
	ST	− 3.41	1.96	− 9.1	− 1.54	1	5405.83
Cross-effects parameters	LPA_MVPA	− 0.02	0.82	− 1.57	1.68	1	2270.19
	LPA_ST	− 0.68	0.68	− 2.1	0.64	1	4439.61
	MVPA_LPA	0.39	0.86	− 1.38	2.06	1	25,240.28
	MVPA_ST	0.12	0.9	− 1.73	1.82	1	11,317.45
	ST_LPA	− 0.47	0.66	− 1.88	0.79	1	23,068.58
	ST_MVPA	− 0.29	0.84	− 1.99	1.34	1	29,190.89
Diffusion parameters	LPA	1.96	0.53	1.39	3.45	1	4370.99
	MVPA	2.62	0.61	1.62	3.84	1	8328.92
	ST	1.92	0.51	1.3	3.32	1	5415.59
	MVPA_LPA	0.15	0.09	− 0.02	0.32	1	8912.24
	ST_LPA	− 0.01	0.09	− 0.19	0.17	1	4292.36
	ST_MVPA	− 0.32	0.1	− 0.56	− 0.15	1	11,144.99
Between-subject	MVPA_LPA	0.25	0.38	− 0.57	0.84	1	9339
	ST_LPA	− 0.37	0.35	− 0.87	0.45	1	39,710
	ST_MVPA	− 0.51	0.29	− 0.89	0.23	1	39,676

Continuous-time intercept = b coefficient; Est. = mean of the chain; BCI = Bayesian credibility interval; Rhat ($\widehat{R}$) = potential scale reduction factor; N_eff_ = effective sample size; LPA = light physical activity; MVPA = moderate-vigorous physical activity; ST = stationary time

**Fig. 1:**
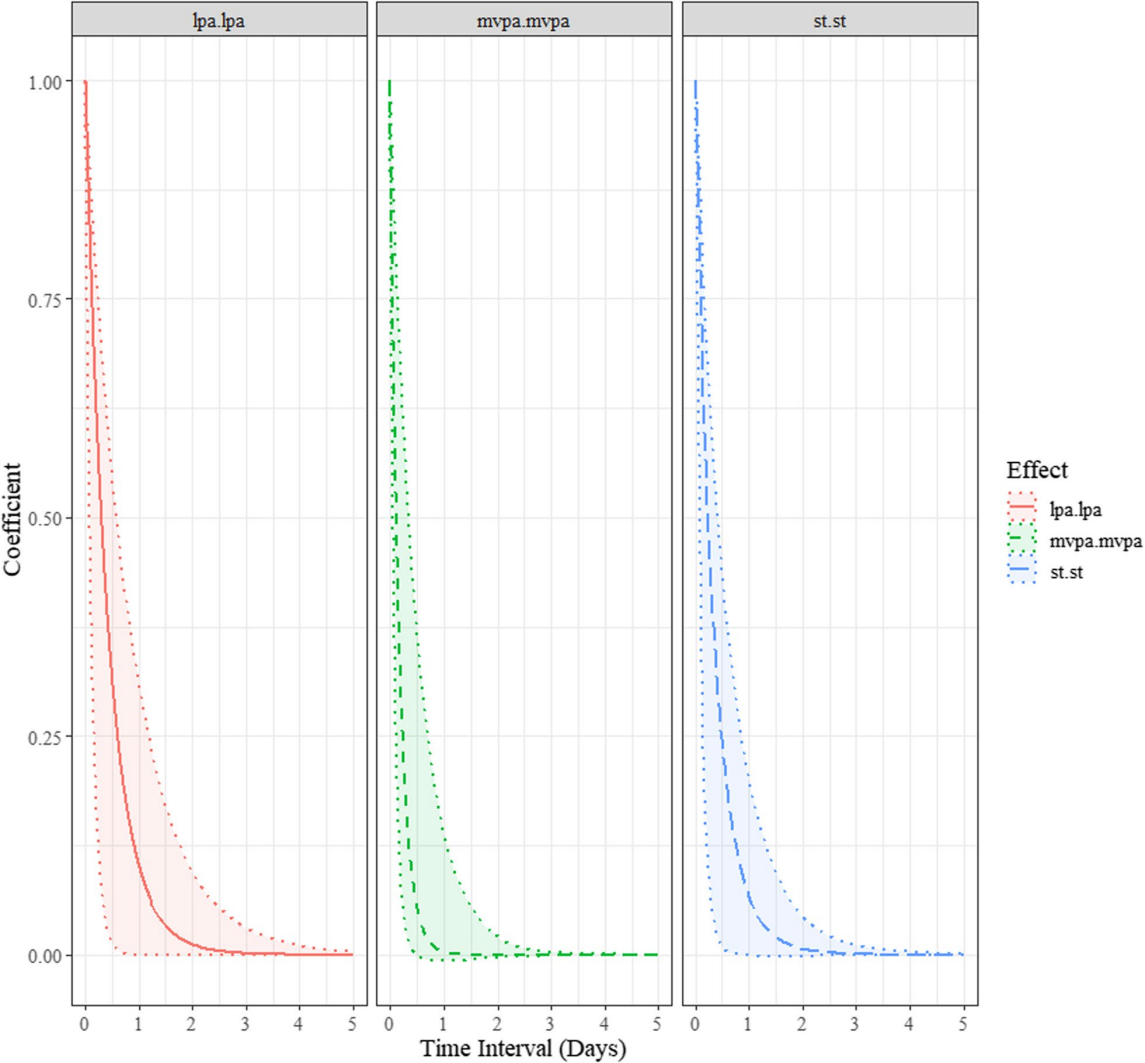
Standardized discrete-time autoregressive effects. LPA = light physical activity; MVPA = moderate-vigorous physical activity; ST = stationary time

**Fig. 2:**
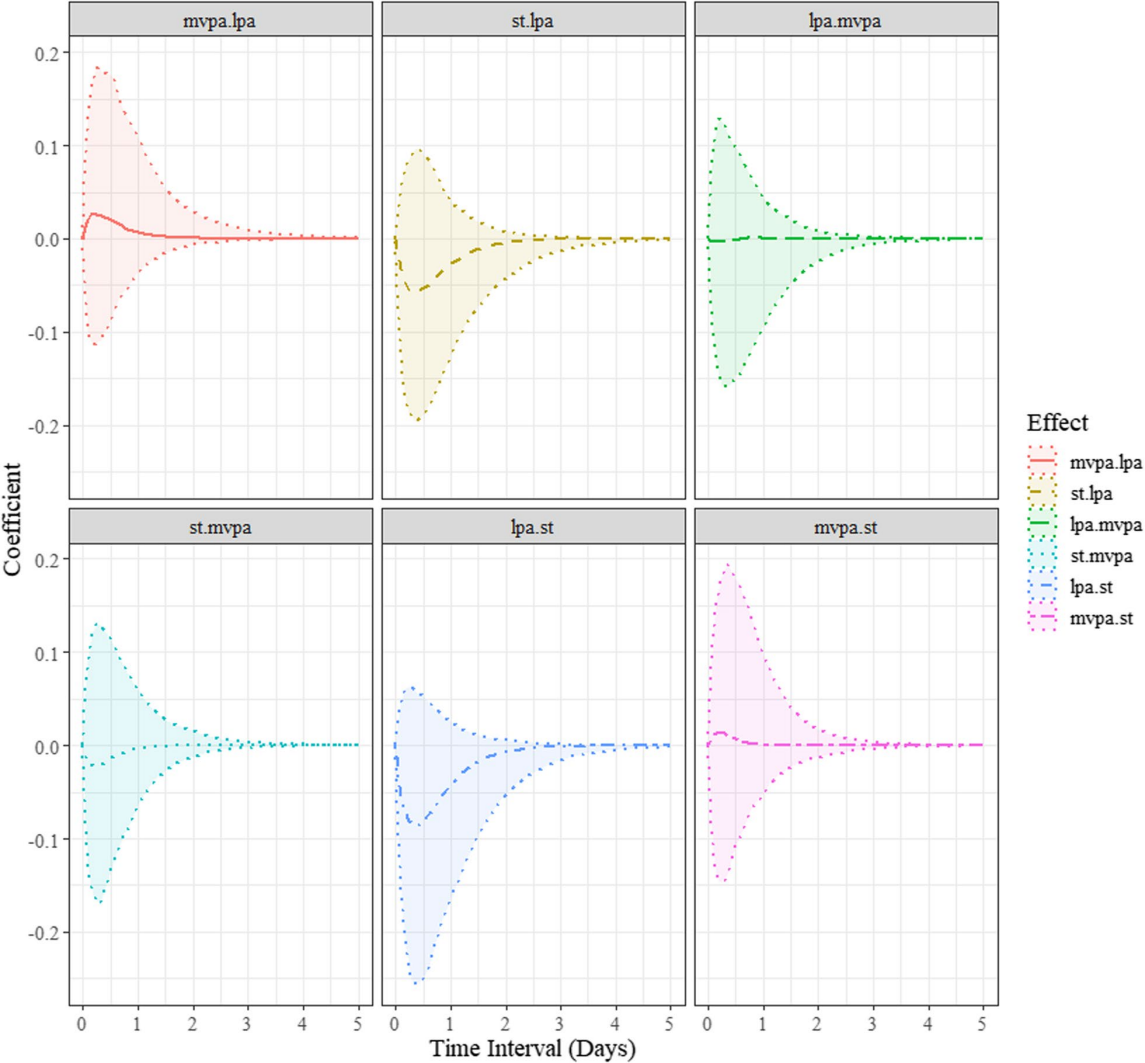
Standardized discrete-time cross-lagged effects. LPA = light physical activity; MVPA = moderate-vigorous physical activity; ST = stationary time

The direct instantaneous (∆*t* → 0) temporal relationship a variable has with its own rate of change (i.e., auto-effects) and between two distinct variables (i.e., cross-effect) are shown in Table 1. These are called drift parameters and both the auto- and cross-effects are of particular interest in this study. For the auto-effects, the closer the estimates are to zero, the longer the changes persisted over time [16, 17]. A negative estimate reveals a diminishing auto-effect over time (returning to baseline) [16, 17]. On the contrary, a positive estimate represents an explosive process (distancing away from the baseline over time). As shown in Table 1 and Fig. 1, the auto-effects for LPA (M = − 3.13, SD = 2, 95% BCI [− 9.04, − 1.24]), MVPA (M = − 6.11, SD = 2.79, 95% BCI [− 12.23, − 1.95]), and ST (M = − 3.41, SD = 1.96, 95% BCI [− 9.1, − 1.54]) demonstrated some degree of persistence, with all the auto-effects parameters excluding zero. LPA, MVPA, and ST were predictors of themselves at a later point in time. For the cross-effects, a negative estimate indicates that an increase in the level of one process predicts a decrease in the level of the other process. A positive cross-effect, in contrast, indicates that an increase in the level of one process predicts an increase in the other process [16, 17]. As demonstrated in Table 1 and Fig. 2, the cross-effect parameters reveal that increases in the levels of LPA, MVPA, and ST do not predict any subsequent change in the levels of the other constructs over time.

The diffusion parameters allow further interpretation of the temporal relationship between the processes (see Table 1). These parameters account for fluctuations in dynamic processes of interest over time that cannot be accounted by the deterministic model elements (i.e., T_0_ mean, drift matrix parameters). The on-diagonal elements of the diffusion matrix are variances that quantify the extent to which the variables in a dynamic system are influenced by unmodelled exogenous inputs [16, 17]. The findings indicate that all latent processes (i.e., LPA, MVPA, ST) were influenced by random (i.e., unpredictable) fluctuations over time. More specifically, LPA and ST processes have similar levels of variation from exogenous inputs (LPA, M = 1.96, SD = 0.53, 95% BCI [1.39, 3.45]; ST, M = 1.92, SD = 0.51, 95% BCI [1.3, 3.32]). As for MVPA, on the contrary, there is relatively more variation in this behaviour over time due to unmodeled factors (M = 2.62, SD = 0.61, 95% BCI [1.62, 3.84]). The off-diagonal elements of the diffusion matrix represent covariances that quantify the extent to which the stochastic variation between two latent processes share common causes [16, 17]. The findings show that there is meaningful negative covariation between ST and MVPA (M = − 0.32, SD = 0.1, 95% BCI [− 0.56, − 0.15]). Furthermore, the 95% BCI for LPA and both MVPA and ST included zero, indicating that variation in LPA is largely independent from the exogenous inputs that influence MVPA (M = 0.15, SD = 0.09, 95% BCI [− 0.02, 0.32]) and ST (M = − 0.01, SD = 0.09, 95% BCI [− 0.19, 0.17]) latent processes. Thus, other factors may determine LPA in this sample.

As illustrated in Fig. 1, the autoregressive effects (i.e., the discrete-time parameters presented in the plots) for children’s LPA, MVPA and ST appear to be predictive of their later behaviours until about 0.5 days later. For example, after accounting for all other dynamic relationships included in the multivariate dynamic model, a child’s MVPA at one moment in time was predictive of their subsequent MVPA until about 12 h hence. This pattern was also consistent across LPA and ST. Further, the autoregressive coefficients are positive (see Fig. 1), demonstrating persistence in processes. In contrast, a negative autoregressive effect illustrates an oscillating compensatory process [14], which was not detected in this study. Further, although no meaningful discrete-time cross-lagged effects were observed over time, Fig. 2 suggests the temporal dependencies among the behaviours were largest at 0.25 days between measurement occasions after accounting for all other dynamic relationships included in the model.

The between-person relationships among LPA, MVPA, and ST are presented in Table 1. No meaningful relationship was found between the behaviours suggesting that the constructs were independent from each other.

## Discussion

The primary aim of this study was to examine the presence of continuous-time multivariate relationships between PA levels and ST in preschool-aged children. Based on the ActivityStat hypothesis, compensatory mechanisms would be indicated by meaningful negative within-behavior relationships for LPA, MVPA or ST [5]. As for the cross-behavioral relationships, ActivityStat is operating when meaningful negative associations are observed between MVPA and LPA, and meaningful positive associations are observed between LPA and ST or between MVPA and ST [5]. Findings for the primary research question show that children with high levels of LPA, MVPA, and ST on a given day, tended to engage in more levels of LPA, MVPA, and ST the following day, respectively. However, the cross-effects parameters indicated no relationship among these behaviours. Thus, this study does not support the ActivityStat in preschool-aged children.

Absent of compensation, some studies have demonstrated similar patterns to what was found in the present study where an increase in PA in a specific context stimulated children between 8 and 10 years of age to engage in more PA at another time [33–35]. Similarly, Nigg et al. [11] report that youth between 6 and 17 years of age do not compensate when MVPA increases. In contrast, a systematic review of 12 studies in preschool-aged children (3–6 years old) found evidence of compensation in 42% of the cases [10], but the studies were not designed explicitly to test for the ActivityStat hypothesis. This fact may have impacted why compensation was not observed [36]. Regardless, investigation of compensatory mechanisms in young children is still understudied and all studies supporting and refuting the ActivityStat hypothesis in this population have employed traditional multilevel models [8, 11, 37], disregarding the temporal multivariate dependence among LPA, MVPA, and ST. Therefore, more studies designed to test the hypothesis are needed in the population of preschool-aged children. Additionally, the use of dynamic modelling methods is critical for understanding such complex systems in future research.

Further interpretation of the temporal relationship between the processes studied show that LPA, MVPA, and ST appear to be influenced by other factors not included in the analysis. For instance, sleep may influence PA and SB in preschool-aged children [1]. This behaviour should be incorporated as a component of the movement behaviour continuum (i.e., sleep, SB, PA) [23]. Poor sleep may increase sedentary-related playtime activities in children while decreasing their MVPA levels [38]. In contrast, variability in LPA appears to have unique causes independent of those that explain variability in MVPA and ST. For example, young children who have siblings may still engage in LPA regardless of restrictions for using outdoor spaces [39]. Due to the complexity of the factors that impact movement-related behaviours, investigating the ActivityStat is a challenge. Future studies should focus on compensatory mechanisms in young children examining simultaneously movement-related behaviours objectively and parents’ perceptions of activity compensation.

The secondary aim of this study was to explore the temporal specificity underlying the multivariate relationships between PA levels and ST. All behaviour autoregressive components were predictive of themselves until about 12 h later. This implies that preschool-aged children who move more at a specific time point subsequently engage in more PA (i.e., LPA and MVPA) until about 12 h later. The same pattern is observed for ST. As for the cross-lagged effects, a relationship among LPA, MVPA, and ST appeared to occur about 0.25 days later. For instance, preschool-aged children who engage in MVPA at a specific time point may engage in more LPA until about 0.25 days later. However, the cross-behavioural relationships among the latent processes were not meaningful and were mostly in the opposite direction of what would have been anticipated by the ActivityStat hypothesis [5]. Thus, no compensation effects were observed at days greater than or equal to one. According to Gomersall et al. [5], the timeframe for compensation is unlikely to occur within a short timescale (i.e., within- and between-days). However, cross-sectional studies testing the hypothesis in children demonstrated evidence of behavioural compensation within- and between-days refuting the argument for longer-term observations [8, 13, 37]. These studies focused on short-time relationships among PA levels and SB and employed traditional longitudinal analyses. Recent systematic reviews highlight the importance of examining the timeframe for compensation as it impacts conclusions of whether compensation occurs [10, 36]. Therefore, because the timeframe for behavioural compensation is unknown, future studies should consider a wider range of timescales, as effects can be aggregated to longer time intervals if required. This potentially has practical importance for designing PA programs impacting on the design, frequency of measurements, and duration of the intervention [5, 10, 36].

This study has some limitations that should be considered when interpreting the results. First, because CT-SEM is computationally intensive (it needs a processor with as many cores as chains, i.e., 4 cores), the dynamic measurement parameters were not included in the final model. Though, this parameter accounts for measurement error in each individual behaviour, it would require a few months for the model to run. Instead, a final model without this parameter still showed adequate model convergence and precision. Additionally, because CT-SEM can accommodate two to three dynamic processes at most, the examination of a complex and multivariate system is computationally burdensome. Therefore, the inclusion of sleep in this study was not feasible. Secondly, the non-experimental design has low internal validity and constrains our ability to make causal claims. Because habitual activity patterns may show temporal variations in PA and ST, these can be misinterpreted as compensatory mechanisms. Additionally, the examination of behavioral compensation in preschool-aged children may be more challenging due to the characteristics of the population. For this age-group, it is difficult to conclude whether variations in PA are due to biological mechanisms, or a response influenced by external factors (e.g., parents, preschool or daycare schedule). Thirdly, SB was represented as ST because the ActiGraph wGT3X-BT detects motion and is limited in reliably detecting the posture component of SB [17, 18]. Lastly, because compensation could occur via energy expenditure mechanisms that were not necessarily reflected in behavioural adaptations [4–6], the lack of energy expenditure measures in this study limited the ability to comprehensively test the hypothesis. Nevertheless, a number of strengths should be highlighted: the use of the novel insights provided by Bayesian CT-SEM to examine the ActivityStat hypothesis, the continuous analysis of processes over time without restricting the timeframe for potential compensation, employing device-assessed measurement of PA and ST over several days, and the examination of this hypothesis in preschool-aged children.

## Conclusion

This is the first study to investigate the ActivityStat hypothesis using Bayesian CT-SEM in preschool-aged children. Though we found positive continuous-time relationships within LPA, MVPA, and ST, no relationships were observed between the behaviours. Overall, the findings do not support the ActivityStat hypothesis in preschool-aged children when simultaneously taking into account all dynamic relationships among LPA, MVPA and ST. One potential explanation is that our participants did not exceed their individual activity set-point as a result of engaging in PA. Therefore, compensatory behaviours were not initiated [5]. Future research should employ dynamic modelling analyses in experimental designs and attempt to measure both energy expenditure and movement behaviours. Absent of evidence for compensation effects, PA programs involving young children should continue to follow current guidelines for promoting health movement behaviour.

## Data Availability

The datasets used and analysed during the current study are available from Valerie Carson on reasonable request.
